# Life-threatening hyperkalemia following zoledronic acid infusion for Paget's disease: a case report

**DOI:** 10.1186/1752-1947-5-367

**Published:** 2011-08-12

**Authors:** Eleftheria Panteliou, Neil Young, Morag Naysmith

**Affiliations:** 1 Intensive Care Unit, Department of Critical Care and Anaesthetics, Western General Hospital, Crewe Road South EH4 2XU, Edinburgh, UK

## Abstract

**Introduction:**

Zoledronic acid is a highly effective treatment in Paget's disease for persistent bone pain and prevention of further progression of the disease. The commonest electrolyte abnormality is hypocalcemia. To the best of our knowledge this is the first case of hyperkalemia secondary to zoledronic acid to be published in the world literature. The commonest arrhythmia related to zoledronic acid is atrial fibrillation.

**Case presentation:**

We describe the case of an 80-year-old Caucasian man, with a history of ischemic heart disease, who had an in-hospital cardiac arrest related to hyperkalemia. Increasing potassium levels were noted following his first zoledronic acid infusion for symptomatic control of bone pain secondary to Paget's disease. Our patient suffered a cardiac arrest 10 days following the zoledronic acid infusion. Our patient's biochemistry and electrocardiogram output were monitored until his death 26 days after his cardiac arrest. Our patient developed paroxysmal atrial fibrillation in the post-resuscitation period and there was persistent hyperkalemia that required prolonged treatment with calcium resonium. All other possible causes of hyperkalemia were excluded.

**Conclusion:**

In our patient's case persistent hyperkalemia and life-threatening arrhythmias were associated with use of zoledronic acid. These side effects have not been reported before and the causative mechanism is far from clear as there are no obvious systemic effects of zoledronic acid. The combination of zoledronic acid with predisposing factors such as structural heart disease might account for the clinical picture we witnessed. As a result, electrolyte monitoring should be adopted early in zoledronic acid use. Further studies are required to elucidate the underlying mechanism of hyperkalemia and identify the target group of patients where zoledronic acid can be safely administered. Great caution is advised in patients with underlying heart conditions.

## Introduction

Four million people are affected by Paget's disease worldwide. Zoledronic acid was licensed for the treatment of Paget's disease in the UK in 2005 and is highly effective providing a prolonged remission after a single intravenous infusion. In the HORIZON ('Health Outcomes and Reduced Incidence with Zoledronic acid ONce yearly') study, involving 10,000 patients, flu-like symptoms, atrial fibrillation and transient renal dysfunction were the commonest side effects. Low calcium, phosphate, magnesium and potassium levels are common electrolyte disturbances described in the literature. One death related to hyperkalemia and acute renal failure in an older patient with osteoporosis and bone metastases following his second zoledronate infusion has been reported to the Medicines and Healthcare Products Regulatory Agency (MHRA) [1]. Hyperkalemia is also documented as a rare side effect on the producing company's database. To the best of our knowledge our report is the first published case.

Zoledronic is a very useful treatment for a large number of patients with a variety of conditions. Potentially fatal side effects, which may remain unnoticed as the majority of patients receive their treatment in an out-patient setting, should become more widely known and efforts should be made to identify and monitor high-risk patients.

## Case presentation

**Figure 1:**
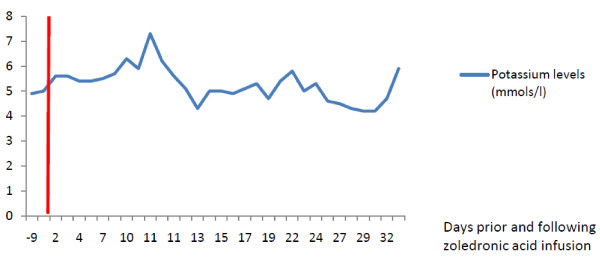
**Potassium levels prior and after zoledronic acid infusion (day 0)**.

An 80-year-old Caucasian man, who was admitted to our facility for symptomatic control of bone pain secondary to Paget's disease, had an in-hospital cardiac arrest related to hyperkalemia following his first zoledronic acid infusion. Despite having had coronary artery bypass grafting for myocardial infarction in the past, there were no ongoing cardiac symptoms. Progressively increasing potassium levels were noted after a single 5 mg zoledronic acid infusion (Figure 1). His renal function remained normal. On admission his potassium level was 4.9 mmol/L (normal range: 3.6 to 5 mmol/L), sodium level 136 mmol/L (normal range 135 to 145 mmol/L), urea level 11.3 mmol/L (normal range 1.7 to 8.3 mmol/L), creatinine level 85μmol/L (normal range 58 to 96μmol/L), estimated glomerular filtration rate (eGFR) >60 mL/minute/1.73 m^2^, calcium level 2.27 mmol/L (normal range 2.1 to 2.6 mmol/L), phosphate level 1.21 mmol/L (normal range 0.8 to 1.4 mmol/L), 25-hydroxyvitamin D level 36 nmol/L (normal range 80 to 150 nmol/L) and alkaline phosphatase level 4973 IU/L (normal range 42 to 128 IU/L). The night prior to his cardiac arrest, his potassium level reached 6.3 mmol/L and our patient received an infusion of insulin and dextrose, as well as calcium gluconate, that resulted in his potassium level being reduced to 5.9 mmol/L. His electrocardiogram results showed bifascicular block and atrial flutter with variable block and a heart rate of 75 beats/minute. His simultaneous calcium level was 2.15 mmol/L and magnesium level was 1.17 mmol/L (normal range 0.7 to 1.0 mmol/L).

Our patient suffered a slow pulseless electrical activity (PEA) cardiac arrest 10 days following the zoledronic acid infusion, lost his cardiac output briefly, received 1 mg of adrenaline and 3 mg of atropine and was resuscitated according to the standard adult life support protocols. His post-resuscitation electrocardiogram showed irregular broad complex tachycardia. His post-cardiac arrest blood results were as follows: potassium 7.3 mmol/L, calcium 2.05 mmol/L (albumin 31 g/L), phosphate 1.3 mmol/L, magnesium 1.17 mmol/L, hydrogen ions 57 nmol/L (normal range 35.5 to 44.5 nmol/L), base excess -8.6 mmol/L (normal range -5 to +3 mmol/L) and lactate 7.8 mmol/L (normal range 0.4 to 2.2 mmol/L). As part of his post-resuscitation care he received a further insulin/dextrose infusion and calcium gluconate.

Following cardiac arrest he maintained his blood pressure and was transferred to the high dependency unit (HDU) for monitoring and further management. Repeat blood results showed potassium 4.3 mmol/L, calcium 1.98 mmol/L, hydrogen ions 38 nmol/L, base excess -1.8 mmol/L, bicarbonate 29 mEq/L (normal range 18 to 23 mEq/L), lactate 3.3 mmol/L, creatinine 59μmol/L, eGFR >60 mL/minute/1.73 m^2^. He was on slow release verapamil for angina (he was β-blocker intolerant because of asthma) that was temporarily discontinued. While in the high-dependency unit he remained in paroxysmal atrial fibrillation/flutter (heart rate 110 to 140 beats/minute). The right bundle branch block noted two weeks prior to the cardiac arrest persisted.

He also developed acute delirium related to hypoxia secondary to pulmonary edema, for which he received non-invasive continuous positive airway pressure respiratory support. His echocardiogram showed mild left ventricular dysfunction, dilated atria and mild pulmonary and tricuspid regurgitation with mild pulmonary hypertension. His troponin I level was mildly elevated, which was not considered to be significant.

His medication history was reviewed and the only medication that had recently been administered that could be associated with the above biochemical results was zoledronic acid. All other possible causes of hyperkalemia were excluded (Table 1). Thyroid function and short synacthen test results were normal. After his discharge from the high-dependency unit his potassium was maintained within the normal range on calcium resonium and his hypocalcemia persisted. Verapamil was restarted in increasing doses to control his ventricular rate. Our patient died of pneumonia 26 days after his cardiac arrest. He had paroxysmal atrial fibrillation until his death.

**Table 1:** Causes of hyperkalemia.

Category	Cause
Excessive potassium intake	Potassium-containing dietary supplements, intravenous potassium infusion

Ineffective potassium renal excretion	Renal impairment

Hormonal	Addison's disease, congenital adrenal hyperplasia, aldosterone deficiency, type IV renal tubular acidosis

Intra-cellular potassium release	Rhabdomyolysis, tumor lysis syndrome, blood transfusion, hemolysis, beta blockers, digoxin toxicity, low insulin levels

Medications	Angiotensin-converting enzyme inhibitors, amiloride, spironolactone, non-steroidal anti-inflammatory drugs, ciclosporin, tacrolimus, trimethoprim, pentamidine, heparin

Pseudohyperkalemia	Hemolysis during venipuncture, thrombocytosis, leukocytosis, polycythemia

## Discussion

Zolendronic acid is a bisphosphonate used in patients with bone metastases from solid tumors, osteolytic lesions in multiple myeloma, osteoporosis and Paget's disease [2]. Zoledronic acid preferentially accumulates in bone and is excreted unchanged in the urine with a half-life of 146 hours [3]. It causes disruption of the 3-hydroxy-3-methyl-glutaryl coenzyme A (HMG-CoA) reductase pathway that is essential for the production of lipid-anchoring cell membrane proteins. Cholesterol metabolites participate in the signaling pathway for interleukin-6 (IL-6)-mediated inflammation [4]. Elevation of tumor necrosis factor and IL-6 occurs one to two days following intravenous administration of bisphosphonates [5] and can increase the incidence of atrial fibrillation [6]. Atrial fibrillation is more common with alendronate in patients taking statins (HMG-CoA inhibitors) [7]. Black *et al*. reported a statistically significant difference (*P *= 0.003) in the occurrence of serious atrial fibrillation in the zoledronic acid group 9 to 11 days after the infusion compared to placebo, although there was no difference in the incidence of all types of atrial fibrillation between the two groups [8]. In the HORIZON trial, involving post-menopausal women with osteoporosis, atrial fibrillation occurred more than 30 days after the infusion. Our patient developed bifascicular block and atrial flutter prior to his cardiac arrest and paroxysmal atrial fibrillation and flutter in the post-resuscitation period. Atrial remodeling and fibrosis have been suggested as likely mechanisms for the development of atrial fibrillation after bisphosphonate administration [9]. In our case no arrhythmia had been clinically diagnosed prior to the infusion, despite the history of ischemic heart disease.

Niemann and Cairns showed that successfully resuscitated animals do not exhibit electrolyte abnormalities [10] and as a result the increased potassium level in our case is thought to be the cause and not the result of the cardiac arrest.

## Conclusion

Zoledronic acid has unknown side effects, despite having been tested prior to its wide clinical use. In our patient persistent hyperkalemia and life-threatening arrhythmias were associated with zoledronic acid infusion. These side effects have not been previously published and the causative mechanism is unclear, as zoledronic acid appears to be bone specific with no obvious systemic effects. In our case hyperkalemia was an early side effect that persisted for approximately four weeks. Atrial fibrillation is a side effect documented in the literature that was also present in our case. Zoledronic acid in relation to ischemic heart disease and structural heart abnormality might be more commonly associated with the clinical picture we observed. Heckbert *et al*. stated that there was no difference in the risk of developing atrial fibrillation with alendronate in the subgroup with history of cardiovascular disease [7]. When zoledronic acid is used, close monitoring of electrolytes is recommended. The correct time for initiating the monitoring and its duration are uncertain. Further investigation as to the mechanisms underlying the development of hyperkalemia, and arrhythmias in association with zoledronic acid would be of benefit. Caution would seem necessary in using zoledronic acid in patients with known heart disease.

## Consent

Written informed consent was obtained from the patient (prior to his death) for publication of this case report and any accompanying images. A copy of the written consent is available for review by the Editor-in-Chief of this journal.

## Competing interests

The authors declare that they have no competing interests.

## Authors' contributions

EP collected the clinical data, reviewed the literature on the topic, and drafted the manuscript. NY and MN reviewed and critically appraised the manuscript. All authors have read and approved the final manuscript.

## References

[B1] UK Medicines and Healthcare Products Regulatory Agency Intravenous zoledronic acid: adverse effects on renal function http://www.mhra.gov.uk/Safetyinformation/DrugSafetyUpdate/CON087704

[B2] Van Beek E Cohen L Leroy I Ebetino F Löwik C Papadopoulos S Differentiating the mechanisms of antiresorptive action of nitrogen containing bisphosphonates Bone 2003 33 805 811 10.1016/j.bone.2003.07.007 14623056

[B3] Chen T Berenson J Vescio R Swift R Gilchick A Goodin S LoRusso S Ma P Ravera C Deckert F Schran H Seaman J Skerjanec A Pharmakokinetics and pharmacodynamics of zoledronic acid in cancer patients with bone metastases J Clin Pharmacol 2002 42 1228 1236 10.1177/009127002762491316 12412821

[B4] Omoigui S Cholesterol synthesis is the trigger and isoprenoid dependent interleukin-6 mediated inflammation is the common causative factor and therapeutic target for atherosclerotic vascular disease and age-related disorders including osteoporosis and type 2 diabetes Med Hypotheses 2005 65 559 569 10.1016/j.mehy.2005.03.012 15935563

[B5] Dicuonzo G Vincenzi B Santini D Avvisati G Rocci L Battistoni F Gavasci M Borzomati D Coppola R Tonini G Fever after zoledronic acid administration is due to increase in TNF- and IL-6 J Interferon Cytokine Res 2003 23 649 654 10.1089/107999003322558782 14651779

[B6] Marcus GM Whooley MA Glidden DV Pawlikowska L Zaroff JG Olgin JE Interleukin 6 and atrial fibrillation in patients with coronary artery disease: data from the Heart and Soul Study Am Heart J 2008 155 303 309 10.1016/j.ahj.2007.09.006 18215601 PMC2247366

[B7] Heckbert SR Li G Cummings SR Smith NL Psaty BM Use of alendronate and risk of incident atrial fibrillation in women Arch Intern Med 2008 168 826 831 10.1001/archinte.168.8.826 18443257

[B8] Black DM Delmas PD Eastell R Reid IR Boonen S Cauley JA Cosman F Lakatos P Chung Leung P Man Z Mautalen C Mesenbrink P Hu H Caminis J Tong K Rosario-Jansen T Krasnow J Hue TF Sellmeyer D Eriksen EF Cummings SR Once-yearly zoledronic acid for treatment of postmenopausal osteoporosis New Engl J Med 2007 356 1809 1822 10.1056/NEJMoa067312 17476007

[B9] Boos CJ Anderson RA Lip GY Is atrial fibrillation an inflammatory disorder? Eur Heart J 2006 27 136 149 16278230 10.1093/eurheartj/ehi645

[B10] Niemann J Cairns C Hyperkalemia and ionized hypocalcemia during cardiac arrest and resuscitation: possible culprits for postcountershock arrhythmias? Ann Emerg Med 1999 34 1 7 10.1016/S0196-0644(99)70265-9 10381988

